# RESOURCES AND EXPERIENCES AMONG DIVERSE DEMENTIA CAREGIVERS BY GEOGRAPHIC CONTEXT

**DOI:** 10.1093/geroni/igac059.2099

**Published:** 2022-12-20

**Authors:** Aya Yoshikawa, Erin Bouldin, Mónika López-Anuarbe, Tiffany Kindratt, Dominique Sylvers, Noah Webster

**Affiliations:** Texas Woman's University, Denton, Texas, United States; University of Utah, Salt Lake City, Utah, United States; Connecticut College, New London, Connecticut, United States; The University of Texas at Arlington, Arlington, Texas, United States; University of Michigan, Ann Arbor, Michigan, United States; University of Michigan, Ann Arbor, Michigan, United States

## Abstract

Rural caregivers are often underserved by caregiving services, yet little is known about how the intersectionality of geographic context and race/ethnicity relates to caregiving resources among dementia caregivers. We examined whether 1) caregiving resources and experiences differ across metro and non-metro areas; and 2) the use of caregiving resources is associated with geographic context by race/ethnicity, controlling for age, gender, and education. We analyzed a sample of caregivers of care recipients aged 65 years or older with ‘probable’ dementia (n= 808) in the 2017 National Health and Aging Trends Study (NHATS) and the associated National Study of Caregiving (NSOC). We defined geographic context by the recipient’s residence in metro (urban) or non-metro (rural) counties and grouped formal (respite care, support groups, caregiving training) and informal (family or friend help) resources. Among minority caregivers, 47% of those living in metro and 36% in non-metro areas used a formal service, and 83% and 72%, respectively, used informal resources. Among White caregivers, estimates were 44%, 48%, 76%, and 66%, respectively. Multivariate regression analyses revealed that non-metro White dementia caregivers had 2.04 times higher odds (95% CI=1.10-3.78) of using formal resources than metro White dementia caregivers. This pattern was not observed among minority dementia caregivers. The use of informal resources did not differ across geographic contexts by race/ethnicity. Findings suggest the influence of geographic context on the use of formal caregiving resources varies by race/ethnicity. With higher rates of dementia in non-metro areas, formal caregiving resources among non-metro minority dementia caregivers need more attention.

